# The site-symmetry induced representations of layer groups on the Bilbao Crystallographic Server

**DOI:** 10.1107/S1600576719011725

**Published:** 2019-10-04

**Authors:** Gemma de la Flor, Danel Orobengoa, Robert A. Evarestov, Yuri E. Kitaev, Emre Tasci, Mois I. Aroyo

**Affiliations:** a Karlsruhe Institute of Technology, Institute of Applied Geosciences, Karlsruhe, Germany; b Departamento de Física de la Materia Condensada, Facultad de Ciencia y Tecnología, Universidad del País Vasco (UPV/EHU), Bilbao, Spain; c Institute of Chemistry, Saint Petersburg State University, Saint Petersburg, Russian Federation; d Ioffe Institute, Saint Petersburg, Russian Federation; e Department of Physics Engineering, Hacettepe University, Ankara, Turkey

**Keywords:** site-symmetry method, *LSITESYM*, Bilbao Crystallographic Server, layer groups

## Abstract

The section of the Bilbao Crystallographic Server (http://www.cryst.ehu.es) dedicated to subperiodic groups includes a new tool called *LSITESYM* for the study of materials with layer and multilayer symmetry. This new program, based on the site-symmetry approach, establishes the symmetry relations between localized and extended crystal states using representations of layer groups. The efficiency and utility of the program *LSITESYM* is demonstrated by illustrative examples, which include the analysis of phonon symmetry in Aurivillius compounds and in van der Waals layered crystals MoS_2_ and WS_2_.

## Abstract 2

The program *LSITESYM* of the Bilbao Crystallographic Server (http://www.cryst.ehu.es), which establishes symmetry relations between localized and extended states in crystals with layer symmetry, is presented. The utility of the program is discussed and illustrated with several examples.

## 1. Introduction   

The interest in layered and multilayered materials such as graphene (Geim & Novoselov, 2007 ▸; Randviir *et al.*, 2014 ▸) and van der Waals crystals, *e.g.* the transition metal dichalcogenide crystal family Me*X*_2_ with Me = Nb, Mo, Ta, W, Ti, V, Zr, Hf and *X* = S, Se, Te (Han *et al.*, 2018 ▸; Manzeli *et al.*, 2017 ▸; Choi *et al.*, 2017 ▸), is constantly growing owing to their interesting properties and possible technological applications. The symmetry of single monolayers can be described by the ‘diperiodic’ or ‘layer groups’ (Kopský & Litvin, 2010 ▸), which are three-dimensional crystallographic groups with two-dimensional translations. There are 80 layer groups which, together with the seven frieze groups (two-dimensional groups with one-dimensional translations) and the 75 rod groups (three-dimensional groups with one-dimensional translations), constitute the ‘subperiodic groups’. The crystallographic data for subperiodic groups are compiled in *International Tables for Crystallography*, Volume E, *Sub­periodic Groups* (hereafter referred to as *ITE*) and also offered online by the Bilbao Crystallographic Server (Aroyo *et al.*, 2011 ▸; Tasci *et al.*, 2012 ▸) (http://www.cryst.ehu.es).

While space groups and their representations describe the symmetry of the bulk electron and phonon states, the layer groups are essential for the description of the electronic structure and the surface states of crystals. Depending on the interaction between the layer and the bulk, materials with layer symmetry can be classified into five different types: (i) pure layered systems like free-standing films, with graphene monolayers as a notable example of such films; (ii) single layers in layered crystals which, owing to a weak van der Waals interlayer interaction, can be separated from the bulk; (iii) artificial nanolayers grown on substrates or between two bulk materials where the interaction between the nanolayer and surrounding bulk materials is neglected; (iv) layers (or slabs) which model atomically clean crystal surfaces where the slab interaction with the rest of the semi-infinite crystal is neglected; (v) interfaces between different crystals, including domain walls approximated by atomically clean crystal surfaces.

The site-symmetry approach (Evarestov & Smirnov, 1987 ▸, 1993 ▸; Hatch *et al.*, 1988 ▸; Stokes *et al.*, 1991 ▸; Kovalev, 1993 ▸) is a powerful method which connects the local properties of atoms in crystals with the symmetry of states of the whole crystalline system, *i.e.* it establishes the symmetry relationships between the localized states in the crystal (vibrations of atoms and atomic orbitals) and crystal extended (phonon and electron) states over the entire Brillouin zone. The site-symmetry method is based on the procedure of induction of representations of the crystal space group from the irreducible representations (irreps) of the site-symmetry groups of the constituent units (atoms, clusters and layers) according to which the local excitations are transformed. In this way, the symmetry of phonon, electron, exciton, biexciton *etc.* states can be described by the crystal single- and double-valued representations induced by the irreps of the site-symmetry group. Tables of induced representations were deduced by Evarestov & Smirnov (1987 ▸, 1993 ▸), Hatch *et al.* (1988 ▸), Stokes *et al.* (1991 ▸) and Kovalev (1993 ▸), and later the programs *SITESYM* and *DSITESYM* (Elcoro *et al.*, 2017 ▸) available on the Bilbao Crystallographic Server were developed to calculate the site-symmetry induced representations of space and double groups, respectively.

Even though layer groups were described for the first time about a century ago (Weber, 1929 ▸; Alexander & Hermann, 1929 ▸), it was not until the paper by Zallen *et al.* (1971 ▸) that they were applied for the first time for the description of phonon states in layered As_2_S_3_ and As_2_Se_3_ crystals. Afterwards, they were also used for modelling atomically clean crystal surfaces (Ipatova & Kitaev, 1985 ▸). However, these studies did not involve the use of induced representations of layer groups. To the best of our knowledge, the first use of induced representations of layer groups was related to the study of the phonon symmetry of high-temperature superconductors (Evarestov *et al.*, 1993 ▸; Kitaev *et al.*, 1994 ▸) and thereafter to describe the phonon, electron, exciton and biexciton states in artificially grown nanolayers (semiconductor quantum wells) (Tronc & Kitaev, 2001 ▸) and layered crystals (Kitaev *et al.*, 2007 ▸). Quite recently, induced representations of layer groups have been applied in the analysis of the Brillouin-zone centre phonons in layered MoS_2_ and WS_2_ crystals (Evarestov *et al.*, 2017 ▸, 2018 ▸).

Note that the induced representations of layer groups, in general, could be extracted by the existing tools of the Bilbao Crystallographic Server for induced representations of space groups, like *SITESYM*, but this procedure would be more complex and prone to errors due to the essential differences between space and layer groups (*e.g.* differences between the sets of Wyckoff positions and their labelling schemes, between the sets of representations *etc.*). The aim of this paper is to present the site-symmetry approach applied to layer symmetry groups for the study of materials with layer symmetry. On the basis of this method, the program *LSITESYM* has been developed and implemented on the Bilbao Crystallographic Server. In the following sections, the procedure of the program for the construction of the induced representations of layer groups is described in detail, and its utility is demonstrated by several examples.

## 2. Site-symmetry method   

### 2.1. General procedure   

The site-symmetry approach establishes the symmetry relations between the crystal extended states induced by localized states of some of the constituent structural units. The procedure for the determination of such a relationship is very useful, as it allows the prediction of the symmetry of the possible extended states starting from the crystal structural data. This task requires the derivation of the irreps of a space group 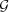 at any point in reciprocal space (which classify the extended states of the structure) induced by the irreps of the site-symmetry group of a Wyckoff position (according to which localized states are classified). In group-theoretical terms, the procedure relating localized and extended crystalline states can be described by induction of a representation of a space group 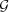 from the irreps of a finite subgroup 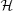, followed by its reduction into irreps of 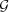. In other words, the induction method permits the calculation of the symmetry of the compatible extended states transforming according to irreps of crystal space group 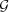 induced by a localized state described by an irrep of the local or site-symmetry group 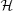 = 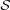. The induction procedure can be applied to any group–subgroup pair 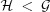, but in the site-symmetry approach, as its name suggests, a site-symmetry group 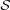 is taken as the subgroup of 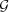. The calculation of the space-group irreps induced by the irreps of a site-symmetry group is not straightforward, because the site-symmetry group 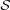 (isomorphic with a point group) is a subgroup of infinite index of 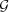. This implies that the representation of 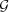 induced by an irrep of 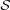 must be of infinite dimension and therefore difficult to calculate directly. This problem is solved by applying the ‘Frobenius reciprocity theorem’ (Serre, 1977 ▸), which states that the multiplicities of the irreps of a group 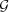 in the induced representation from an irrep of a subgroup 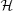 of 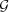 can be determined from the multiplicities of the irreps of 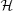 in the representations subduced from 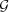 to 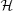. Therefore, the site-symmetry method is based on subduction and induction, two basic concepts of representation theory, and on the Frobenius reciprocity theorem.

The subduction procedure relates the representations of a group 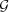 to those of its subgroups 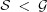. Consider an irrep **D**_γ_ = 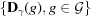 of a group 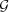. The subduction of **D**_γ_ to the subgroup 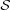 results in a representation of the subgroup, known as the ‘subduced representation’ **D** ^Sub^, formed by the matrices of those elements of 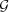 that also belong to the subgroup 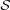, *i.e.* **D** ^Sub^ = 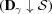 = 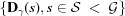. This subduced representation 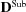 is in general reducible and is decomposable into irreps 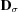 of 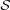:
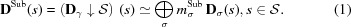
The multiplicities 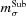 of the irreps 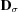 of 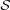 in the subduced representation 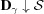 can be calculated by the reduction formula (known also as the ‘magic’ formula):
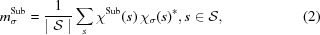
where χ^Sub^(*s*) is the character of the subduced representation and χ_σ_(*s*) is the character of the irrep 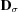 for the same element 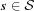.

The induction procedure permits the construction of a representation of 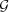 starting from a representation of 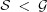. If 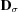 = 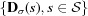 is an irrep of 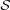, then the matrices of the induced representation 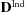 = (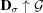) of 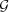 are constructed as follows:
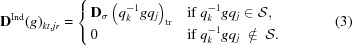
Here, *t*, *r* = 1, …, *m* with *m* equal to the dimension of the irrep 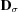 of 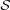 and *k*, *j* = 1, …, *n* where *n* = 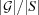 is the index of 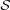 in 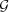. The elements 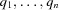 are the coset representatives of the decomposition of 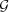 with respect to 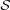.

The characters of 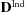 are given by
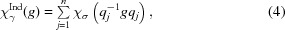
where 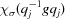 is the trace of the *j*th diagonal block of 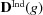 and *n* is the index of the subgroup in the group (which can be finite or infinite). In general, the induced representations are reducible, and as such it is possible to decompose them into irreps 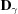 of 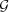:
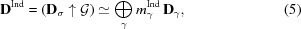
where 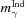 are the multiplicities of the irreps of 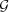 in the induced representations 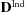 and can be calculated by the reduction formula [see equation (2)].

The dimension of the induced representation can be read directly off the equation for its construction [equation (3)]:
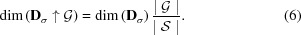
This result points out the difficulties for the direct calculation of a representation of a space group 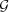 induced from an irrep of a finite subgroup 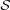 of 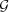. As the site-symmetry group is a subgroup of an infinite index, this suggests that the dimension of the induced irreps must be of infinite dimensions. By means of the site-symmetry approach it is possible to determine the multiplicities 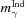 of an irrep of 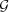 in the induced representation without the necessity of constructing the infinite-dimensional representation. The method is based on the Frobenius reciprocity theorem, according to which the multiplicity of an irreducible irrep 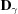 of 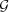 in a representation (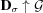) of 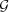 induced by an irrep 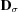 of 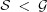 is equal to the multiplicity of the irrep 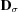 of 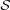 in the representation (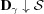) subduced by 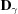 of 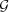 to 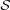, *i.e.* 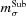 = 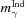. In other words, it is sufficient to calculate the multiplicities 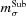 of 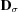 in the subduced representation (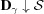) in order to obtain the frequencies 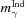 of 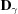 in the induced representation (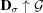).

### 2.2. Application of site-symmetry method to layer groups   

The group–subgroup relations between layer and space groups 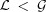 are essential to extend the site-symmetry method to layer groups. These relationships have been considered in detail in the literature [see *e.g.* Wood (1964 ▸), *ITE* and references therein]. The type of space group of which a given layer group is a subgroup is not defined uniquely. The ‘simplest’ space group 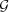 to which 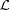 is related can be expressed as a semi-direct product of 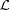 with the one-dimensional translation group *T*_3_ of additional translations 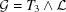, where *T*_3_ is a normal subgroup of 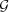. Thus, the layer group 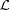 is isomorphic with the factor group 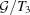.

The isomorphism between 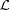 and the factor group 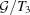 results in close relationships between the Wyckoff positions and the irreps of 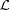 and 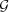. One can show that the set of Wyckoff positions of a layer group is contained in the set of Wyckoff positions of the related space group (*cf.* Evarestov & Smirnov, 1993 ▸). For example, consideration of the restrictions imposed by the loss of periodicity in the third (*z*) direction yields the following restrictions on the special-position coordinates of layer groups: only the special positions of 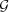 whose *z* coordinate does not involve a fraction of the unit-cell parameter are possible special positions of 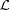, *i.e.* special positions of 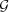 with *z* coordinates *z*, −*z* or 0. In that way, to each Wyckoff position of 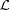 corresponds exactly one Wyckoff position of 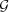, specified by exactly the same site-symmetry group and multiplicity, and by the same set of coordinate triplets of equivalent positions. Thus, the description of the Wyckoff positions of layer groups can follow the Wyckoff-position descriptions used in space groups. Note, however, that according to the conventions adopted in *ITE* the letter labelling of the Wyckoff positions of layer groups is done independently of that of space groups. As a result, the Wyckoff letters of the corresponding space- and layer-group Wyckoff positions might not coincide, in general.

The simple relationship between the irreps of 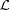 and 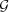 is based on the isomorphism 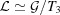: the irreps of 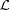 are also irreps of 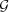 and every irrep of 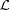 is related to a specific irrep of 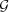. In these irreps of 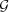 all elements of a given coset of the decomposition of 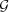 with respect to *T*_3_ are mapped onto the same matrix, *i.e.* the irreps of 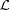 coincide with those irreps of 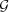 whose kernel is *T*_3_ (for details, see Evarestov & Smirnov, 1993 ▸). For example, the special **k** vectors of 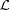 can also be deduced from the **k** vectors of 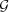. The Brillouin zone of a layer group 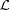 can be described as a projection of the Brillouin zone of the corresponding space group 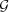 onto the layer plane. Accordingly, the two-dimensional set of **k** vectors **k**(*k*_1_, *k*_2_) of 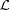 can be obtained from the three-dimensional **k**(*k*_1_, *k*_2_, *k*_3_) vectors of 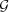 by ignoring the third component *k*_3_.

The above considerations indicate that the induced representations of a layer group can be read off directly from the induced representations of the corresponding space group. On the basis of this observation, the procedure of the site-symmetry method for layer groups can be summarized as follows:

(i) Given the layer group 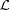 (specified by its number), the Wyckoff position and the **k** vector (*k*_1_, *k*_2_), the program identifies (*a*) the corresponding space-group number and Wyckoff letter, and (*b*) the space-group wave vector (*k*_1_, *k*_2_, *k*_3_) with *k*_3_ = 0, related to the layer-group **k** vector (*k*_1_, *k*_2_).

(ii) The site-symmetry method for space groups is applied, *i.e.* the site-symmetry induced representations are calculated by the program *SITESYM*. [More detailed information on the algorithm of *SITESYM* can be found in the work of Elcoro *et al.* (2017 ▸).]

(iii) The site-symmetry induced representations of space group 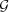 obtained by *SITESYM* are described with respect to the layer group 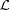.

### 2.3. The program *LSITESYM*   

The computer program *LSITESYM* establishes symmetry relations between localized and extended states in crystals with layer symmetry. This algorithm calculates the multiplicities of the layer-group irreps in the representation induced by the irreps of a site-symmetry group (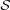 < 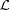).

The necessary input steps of *LSITESYM* and its corresponding output will be illustrated by the study of the symmetry of the phonon states in the Aurivillius compounds [Bi_2_O_2_]^+2^[*A*_*n*−1_ *B*_*n*_O_3*n*+1_]^+2^ (known for their ferroelectric properties), a problem discussed in detail by Kitaev *et al.* (2007 ▸). These compounds exhibit two types of layer symmetry: for even *n*, the layers are described by the layer group *p*4*mm* (No. 55), while for odd *n* there is one central layer with higher symmetry described by the layer group *p*4/*mmm* (No. 61). The output of the program will be illustrated by the specific calculations of the symmetry relationships between the phonon states at the point **k** = *M*(½, ½) of the layer group *p*4/*mmm* and the localized states of atomic orbitals at the Wyckoff position 2*c* (0, ½, 0) with site-symmetry group *mmm.*. The irreps of the layer group *p*4/*mmm* at the point **k** = *M*(½, ½) with nonzero multiplicities, shown in the output of *LSITESYM*, describe the transformation properties of the extended phonon states induced by the irreps of the layer site-symmetry group *mmm.* of the Wyckoff position 2*c* (0, ½, 0).

In the INPUT block of the program the user is expected to provide the layer group, an occupied Wyckoff position with its representative coordinate triplet and the **k** vector (specified by its coordinates and label) of the layer-group irreps **D**_γ_ whose induced-representation multiplicities are to be calculated. The information is entered in three steps: in the first one, the layer group is specified by its *ITE* sequential number; in the second step, the occupied Wyckoff positions are to be selected from a list produced by the program; and finally, the coordinates and the label of the **k** vector of the irreps **D**_γ_ have to be introduced.

The OUTPUT block starts with a header that reproduces the input data, followed by a display of tables with the results of the intermediate steps of the procedure:

#### List of operations of the layer site-symmetry group 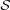   

2.3.1.

Each of the symmetry operations of the layer group that leaves the Wyckoff position representative point invariant is specified by its shorthand description (coordinate triplet) and matrix-column representation. Labels (*g*_1_,…, *g_n_*), necessary for later referencing, are assigned to each element of 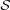.

**Figure 1:**
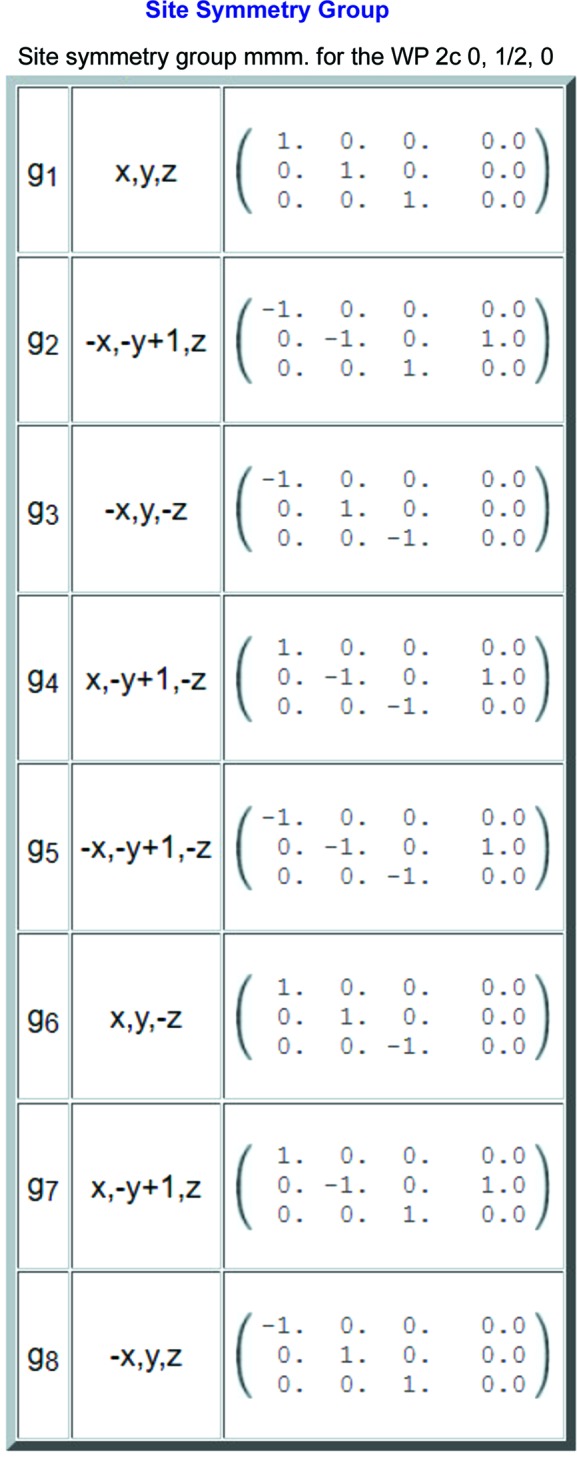
A screenshot of the partial output of the program *LSITESYM*, showing the eight symmetry operations of the site-symmetry group *mmm.* of the Wyckoff position (WP) 2*c* (0, ½, 0) of the layer group *p*4/*mmm* (No. 61). The symmetry operations are specified by their shorthand descriptions (coordinate triplets) and matrix-column representations.

The layer site-symmetry group *mmm.* of the position 2*c* (0, ½, 0) of the layer group *p*4/*mmm* is formed by eight symmetry operations, as shown in the screenshot (Fig. 1 ▸) of the program *LSITESYM*.

#### 2.3.2. Character table of the point group   

This table reproduces the character table of the irreps **D**_σ_ of the point group isomorphic with the site-symmetry group 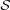. The notations of Mulliken (1933 ▸) and Koster *et al.* (1963 ▸) are applied to label the irreps [see also Bradley & Cracknell (1972 ▸)].

**Figure 2:**
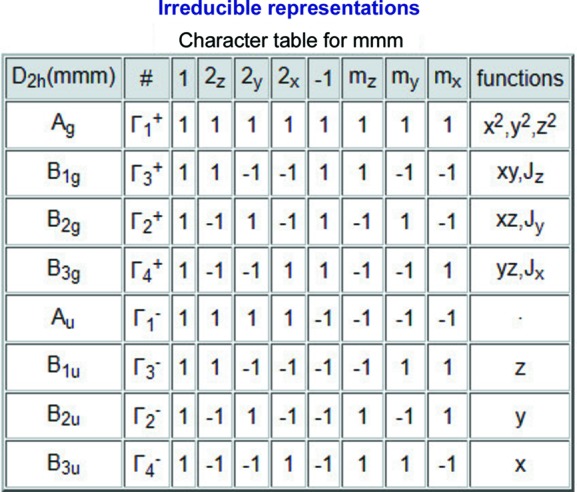
A screenshot of the partial output of the program *LSITESYM*, showing the character table of the point group *mmm*, isomorphic with the site-symmetry group of the Wyckoff position 2*c* (0, ½, 0) of the layer group *p*4/*mmm* (No. 61). The irreps are labelled according to the notation of Mulliken (1933 ▸) and Koster *et al.* (1963 ▸).

The site-symmetry group of the point (0, ½, 0) is isomorphic with the point group *mmm* and has eight irreps. The character table is reproduced in Fig. 2 ▸.

#### 2.3.3. Table of characters of the subduced representations   

The characters of the elements of the site-symmetry group 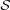 (obtained in the first step) for each of the irreps **D**_γ_ of 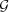 of the selected wavevector are calculated internally by the program *REPRES* (*cf.* Aroyo *et al.*, 2006 ▸). In this way, the characters of the subduced representations (**D**_γ_ ↓ 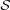) of 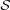 are obtained. The notation of the layer-group irreps has been chosen to be the same as for the corresponding irreps of the related space group, and it follows that of Cracknell *et al.* (1979 ▸).

**Figure 3:**
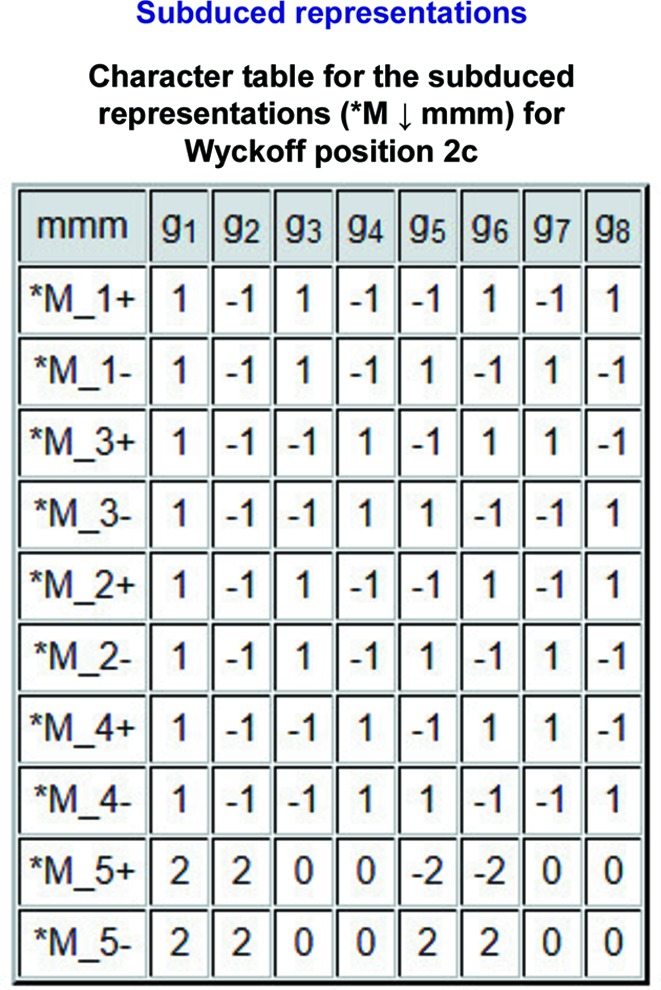
A screenshot of the partial output of the program *LSITESYM*, showing the characters of the subduced representations of the site-symmetry group 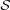 of the Wyckoff position 2*c* (0, ½, 0) of the layer group *p*4/*mmm* (No. 61) at the **k** point *M*. The columns are specified by the symmetry operations of the site-symmetry group 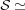 *mmm* (*cf.* Fig. 1 ▸).

The layer group *p*4/*mmm* has ten irreps for the **k** vector *M*. Fig. 3 ▸ shows the characters of the corresponding subduced representations (*M_*i*_ ↓ *mmm*) of the site-symmetry group. In accordance with the notation of the space-group irreps, *M_i_ and *M_i_* denote the full group and the little group irrep of the layer group, respectively.

#### 2.3.4. Table of the decompositions of the subduced representations   

**Figure 4:**
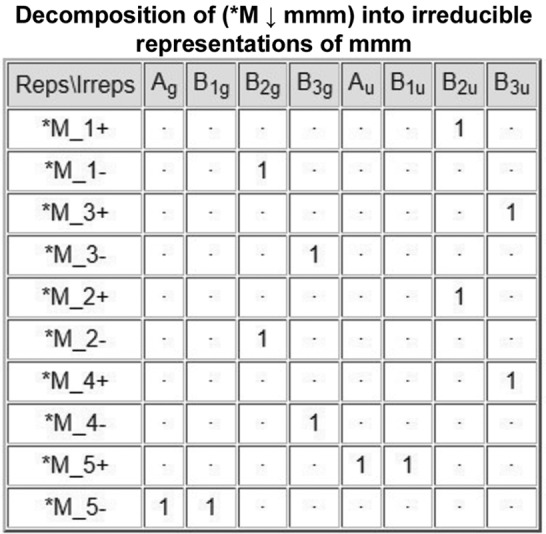
A screenshot of the partial output of the program *LSITESYM*, showing the decompositions of the subduced representations (*M_*i*_ ↓ *mmm*), shown in Fig. 3 ▸, into irreps of *mmm* (*cf.* Fig. 2 ▸).

The multiplicities 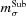 of the irreps of 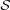 in the subduced representations (**D**_γ_ ↓ 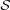) are obtained by the application of the reduction formula [see equation (2)]. In the example, the decompositions of the representations (*M_i_ ↓ *mmm*), *i* = 1, …, 10, into irreps of *mmm* are shown in Fig. 4 ▸.

#### 2.3.5. Table of induced representations   

According to the Frobenius reciprocity theorem, the multiplicities 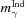 of the irreps **D**_γ_ of 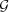 for a given **k** vector in the representations (**D**_σ_ ↑ 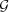) (induced from the irreps **D**_σ_ of the site-symmetry group 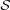) are obtained by transposing the table of the decompositions of the subduced representations (**D**_γ_ ↓ 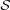).

**Figure 5:**
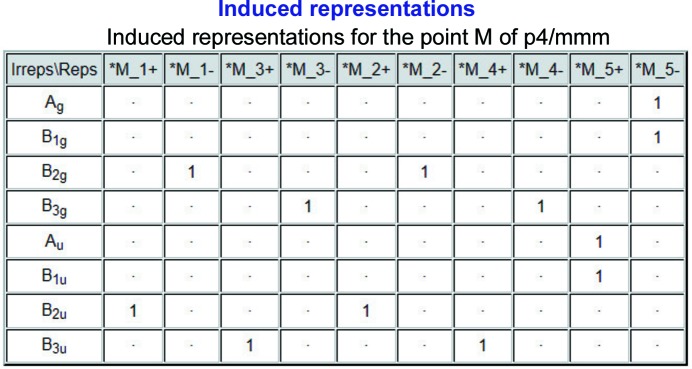
A screenshot of the partial output of the program *LSITESYM*, showing the representations of the layer group *p*4/*mmm* (No. 61) at the point *M* induced by the irreps of the site-symmetry group *mmm*. of the Wyckoff position 2*c* (0, ½, 0).

The table of representations of the layer group *p*4/*mmm* at the point *M* induced by the irreps of the site-symmetry group *mmm*. of the Wyckoff position 2*c* (0, ½, 0) is shown in Fig. 5 ▸. The rows of the table correspond to the irreps **D**_σ_ of the site-symmetry group *mmm*. (*cf.* Fig. 2 ▸); the entries in each row indicate the multiplicities of the *M* irreps of *p*4/*mmm* in the (infinite-dimensional) induced representation (**D**_σ_ ↑ *p*4/*mmm*):
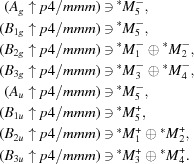
The obtained results coincide exactly with the corresponding data of Table V in the work by Kitaev *et al.* (2007 ▸).

#### 

The URL of the program *LSITESYM* is http://www.cryst.ehu.es/subperiodic/layer_sitesym.html.

## 3. Transition metal dichalcogenide layer crystals   

We now illustrate the use of the *LSITESYM* program described in Section 2, taking as an example MoS_2_ and WS_2_ layered crystals which belong to the transition metal dichalco­genide crystal family. Although the structure of MoS_2_ was determined about 100 years ago and this layered crystal was used mainly as a dry lubricant, the new wave of interest in transition metal dichalcogenides began after the discovery of graphene. This interest, apart from the fundamental properties of monolayers, is connected with the observation of the direct band gap of 1.8 eV at the *K* point in the MoS_2_ monolayer, even though the bulk MoS_2_ crystal is a semiconductor with an indirect band gap of 1.2 eV. The transition to a direct band gap makes the MoS_2_ monolayer an excellent candidate as a solar photovoltaic material owing to the drastic enhancement of photoluminescence in the monolayer compared with the bulk. Other crystals of the transition metal dichalcogenide family, like WS_2_, have similar electronic properties.

Comprehensive reviews and many references to studies of this material can be found in the work of Wang (2014 ▸), Kolobov & Tominaga (2016 ▸) and Manzeli *et al.* (2017 ▸).

**Figure 6:**
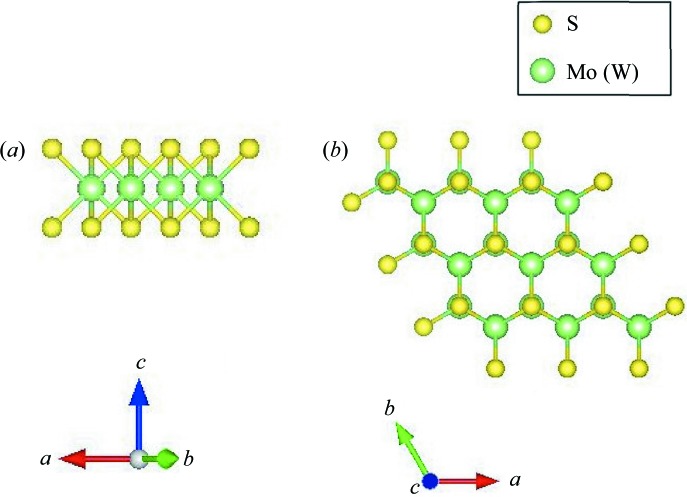
(*a*) The crystal structure of a single layer of MoS_2_ (WS_2_), and (*b*) its projection along [001].

The space group of the MoS_2_ and WS_2_ bulk crystals is *P*6_3_/*mmc* (No. 194). In the bulk crystal, the metal atoms occupy the 2*c* (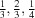) position and the sulfur atoms occupy the 4*f* (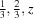) position (Lee *et al.*, 2014 ▸). The layer group of a single layer 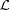 = 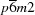 (No. 78) (Milošević *et al.*, 2000 ▸) is isomorphic with the factor group 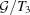, where 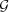 is the space group 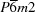 (No. 187), *i.e.* 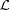 is a subgroup of 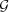. The crystal structure of a single layer is shown in Fig. 6 ▸. Atoms in the primitive unit cell of the layer occupy the following Wyckoff positions: Mo (W) 1*c* (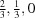), S 2*e* (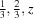). Note that the layer group of a single layer is also a subgroup of the space group of the bulk crystal.

The phonon symmetry in these crystals has been studied by Molina-Sánchez & Wirtz (2011 ▸), Ribeiro-Soares *et al.* (2014 ▸) and Saito *et al.* (2016 ▸). However, diperiodic groups were introduced explicitly only by Evarestov (2015 ▸), Evarestov *et al.* (2017 ▸) and Bocharov *et al.* (2019 ▸). In the work of Ribeiro-Soares *et al.* (2014 ▸), the symmetry of single layers was described in terms of the related space group 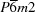 (No. 187) and, in addition, the authors applied a non-standard irrep notation, which hinders the use and comparison of their results.

The results of *LSITESYM*, shown in Table 1 ▸, permit the analysis of the phonon symmetry of a single MoS_2_ layer. Table 1 ▸ is organized as follows. The 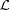 irreps describing the symmetry of phonons at the 2D Brillouin-zone symmetry points are induced by the irreps **D**_σ_ of the site-symmetry groups 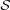 of the Wyckoff positions where the atoms given in column 1 are located. The localized atomic displacements *x*, *y* and *z* transforming according to the irreps **D**_σ_ are indicated in brackets. The labels of layer group 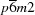 irreps at the **k** vector points of the two-dimensional Brillouin zone coincide with the labels of the irreps of the corresponding points of the three-dimensional Brillouin zone of the related space group 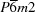.

From Table 1 ▸ it is seen that the Mo atom vibrations along the *z* axis induce Γ_3_ modes, whereas vibrations in the *xy* plane induce Γ_5_ modes. Similarly, S atom vibrations along the *z* axis and in the *xy* plane induce Γ_1_ + Γ_3_ and Γ_5_ + Γ_6_ modes, respectively. The vibrational representation at the Γ point can be written down, summing the contributions of all the atoms in the primitive unit cell. It is given by Γ = Γ_ac_ + Γ_opt_ = (Γ_3_ + Γ_5_) + (Γ_1_ + Γ_3_ + Γ_5_ + Γ_6_), where the subscripts ac and opt indicate acoustic and optical layer modes, respectively. Further, one can establish a correspondence between layer and bulk crystal modes. Acoustic layer modes induce interlayer bulk modes, whereas optical layer modes induce intralayer bulk ones. The phonon symmetry in a bulk MoS_2_ crystal obtained by the *SITESYM* program (Elcoro *et al.*, 2017 ▸) is given in Table 2 ▸. The notations in Table 2 ▸ are the same as those in Table 1 ▸.

Knowing the symmetry of bulk and layer phonons, one can obtain the genesis of the bulk modes from the layer ones. This could be determined using the *CORREL* program of the Bilbao Crystallographic Server (Aroyo *et al.*, 2006 ▸) for the group–subgroup pair, namely *P*6_3_/*mmc* and 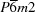. The factor group of the latter is isomorphic with the layer group 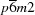. When applying this procedure it is necessary to choose correctly the transformation matrix relating the conventional settings of the group and the subgroup. For the *P*6_3_/*mmc* and 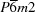 pair, the transformation matrix is reduced to an origin shift (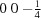).

On the basis of the Frobenius theorem, the correspondence between the layer irreps at the Γ point and the bulk irreps is given in Table 3 ▸. It is seen that each layer mode generates a pair of bulk modes at the Γ point, one odd and one even. The splitting is due to a weak van der Waals interaction between the layers. Therefore, the frequencies of the modes constituting a pair would be close.

Similarly, one can deduce the correspondence between layer and bulk modes at other Brillouin-zone points. For example, the correspondence between the layer (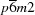) modes at the point 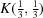 and the bulk (*P*6_3_/*mmc*) modes at 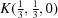 and 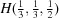 are shown in Table 4 ▸.

## 4. Conclusions   

A new computer tool which calculates the site-symmetry induced representations of layer groups, called *LSITESYM*, has recently been implemented on the Bilbao Crystallographic Server. Like the rest of the programs on the server, this new tool is freely available and can be accessed via user-friendly web interfaces. The algorithm of *LSITESYM*, based on the site-symmetry method applied to layer groups, is an extension of the algorithm used in the program *SITESYM* for ordinary space groups. The group–subgroup relation between layer and space groups 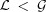 is fundamental for the development of the procedure for layer groups. On the basis of the isomorphism between the layer 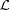 and the factor group 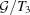, it is possible to establish a simple connection between the Wyckoff positions, **k** vectors and irreps of 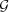 and 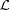, which is essential to calculate the site-symmetry induced representations of layer groups.

The program *LSITESYM*, which is able to determine the symmetry relations between localized and extended states in crystals with layer symmetry, is also very useful in the description of phonon states and electronic structure. The capabilities of the program *LSITESYM* have been successfully demonstrated in several examples. Moreover, the utility of the program in combination with other tools of the Bilbao Crystallographic Server to obtain the relation between bulk and layer modes has also been shown.

## Figures and Tables

**Table 1:** Phonon symmetry in the single MoS_2_ layer with 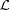 = 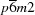 (No. 78)

Atom	Wyckoff position	**D**_σ_	Γ (0, 0) 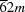	*K* ( 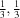 ) 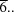	*M* ( 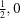 ) *m*2*m*
Mo	1*c*	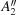 (*z*)	3	6	3
	( 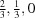 )	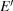 (*x*, *y*)	5	1, 3	1, 2
	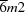				
S	2*e*	*A*_1_ (*z*)	1, 3	3, 4	1, 3
	( 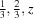 )	*E* (*x*, *y*)	5, 6	1, 2, 5, 6	1, 2, 3, 4
	3*m.*				

**Table 2:** Phonon symmetry in the MoS_2_ bulk crystal *P*6_3_/*mmc* (No. 194)

Atom	Wyckoff position	**D**_σ_	Γ ( 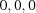 ) 6/*mmm*	*A* ( 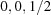 ) 6/*mmm*	*K* ( 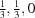 ) 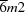	*H* ( 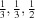 ) 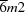	*M* ( 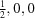 ) *mmm*	*L* ( 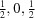 ) *mmm*
Mo	2*c*	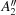 (*z*)	2^−^, 3^+^	1	6	2	2^−^, 3^+^	1
	( 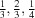 )	*E*′ (*x*, *y*)	5^+^, 6^−^	3	1, 4, 5	2, 3	1^+^, 2^+^, 3^−^, 4^−^	1, 2
	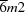							
S	4*f*	*A*_1_ (*z*)	1^+^, 2^−^, 3^+^, 4^−^	1, 1	5, 6	1, 2	1^+^, 2^−^, 3^+^, 4^−^	1, 1
	( 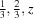 )	*E* (*x*, *y*)	5^+^, 5^−^, 6^+^, 6^−^	3, 3	1, 2, 3, 4, 5, 6	1, 2, 3, 3	1^+^, 1^−^, 2^+^, 2^−^, 3^+^, 3^−^, 4^+^, 4^−^	1, 1, 2, 2
	3*m.*							

**Table 3:** Correspondence between the layer (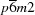) irreps at the point Γ(0, 0) and the bulk (*P*6_3_/*mmc*) irreps at Γ(0, 0, 0) and *A*(½, ½, 0)

Layer		Bulk
Γ_1_	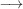	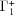 + 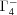 + *A*_1_
Γ_2_	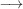	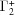 + 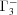 + *A*_2_
Γ_3_	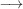	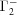 + 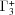 + *A*_1_
Γ_4_	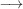	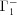 + 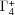 + *A*_2_
Γ_5_	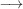	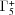 + 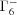 + *A*_3_
Γ_6_	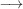	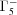 + 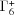 + *A*_3_

**Table 4:** Correspondence between the layer (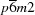) irreps at the point *K*(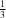, 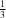) and the bulk (*P*6_3_/*mmc*) irreps at *K*(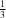, 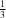, 0) and *H*(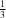, 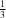, ½)

Layer		Bulk
*K*_1_	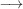	*K*_1_ + *K*_4_ + *H*_3_
*K*_2_	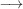	*K*_2_ + *K*_3_ + *H*_3_
*K*_3_	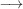	*K*_5_ + *H*_1_
*K*_4_	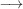	*K*_6_ + *H*_2_
*K*_5_	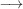	*K*_5_ + *H*_2_
*K*_6_	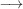	*K*_6_ + *H*_1_
